# Role of magnetic resonance imaging in acute spinal trauma: a pictorial review

**DOI:** 10.1186/s12891-016-1169-6

**Published:** 2016-07-22

**Authors:** Yogesh Kumar, Daichi Hayashi

**Affiliations:** Department of Radiology, Bridgeport Hospital, Yale New Haven Health System, 267 Grant Street, Bridgeport, CT 06610 USA; Department of Radiology, Boston University School of Medicine, 820 Harrison Avenue, FGH Building, 3rd Floor, Boston, MA 02118 USA

**Keywords:** Spinal trauma, MRI, Spinal cord, Hemorrhage, Ligamentous injury

## Abstract

Magnetic resonance imaging (MRI) has been playing an increasingly important role in the spinal trauma patients due to high sensitivity for detection of acute soft tissue and cord injuries. More and more patients are undergoing MRI for spinal trauma in the emergency settings, thus necessitating the interpreting physicians to be familiar with MRI findings in spinal trauma. In this pictorial review, we will first describe the normal anatomy of various ligamentous structures. Indications of MRI in spinal trauma as well as the role of MRI in diagnosing spinal cord and soft tissue injuries will then be discussed. Illustrated cases are mainly of cervical spine trauma, but thoracolumbar spine injuries are also included where appropriate in our review.

## Background

Imaging plays a critical role in diagnosis of acute spinal trauma and helps in initiating prompt and accurate treatment in these patients. Conventional radiographs and computed tomography (CT) are the initial imaging modalities used in the diagnosis of most cases of spinal injuries. While stability of the spine may be adequately assessed with CT for surgical decision making by spine surgeons [1], due to its increased availability in the emergency settings and its inherently superior contrast resolution, MRI has been playing an increasingly important role in the management of spinal trauma patients. Notably, MRI is the modality of choice for evaluation of ligamentous and other soft tissue structures, disc, spinal cord and occult osseous injuries [2]. In this pictorial review, we will first describe the normal anatomy of various ligamentous structures including the craniocervical junction. Then, indications of MRI in spinal trauma as well as the role of MRI in diagnosing spinal cord, soft tissue injuries and occult osseous injuries will be discussed (Table 1). Illustrated cases are mainly of cervical spine trauma, but thoracolumbar spine injuries are also included where appropriate. Various limitations and pitfalls of MRI in spinal trauma imaging will also be discussed.

**Table 1:** Role of MRI for evaluation of various acute traumatic spinal injuries

Pathologic features	Role of MRI
Ligamentous injury	• Higher sensitivity for detection compared to CT. • Complete tear (seen as discontinuity of ligaments) or partial tear (seen as abnormal signal) can be differentiated. • Helpful in guiding management by differentiating stable from unstable injuries.
Disc damages and herniations	• Detection of abnormal disc signal related to traumatic herniations. • Important to diagnose this before closed reduction as undetected disc herniations can cause worsening cord injury.
Extra medullary hemorrhage	• MRI shows extent of hematoma to help in surgical planning. • Extradural hematoma is commonly encountered and can lead to cord compression.
Vascular injuries	• Enable detection of arterial injuries, which include an intimal flap, pseudoaneurysm, complete occlusion or active extravasation. • Undetected vascular injuries can cause spinal cord infarctions.
Cord injuries	• Detection of hemorrhagic and non-hemorrhagic cord injuries. • This is the single most important role of MRI in spinal trauma evaluation. • Visualized as abnormal cord signal with hemorrhage best seen on gradient recalled echo (GRE) type sequences. • Presence of hemorrhage is the most important poor prognostic factor.
Acute vs old vertebral fracture	• Age-indeterminate fractures identified on radiography and CT can be classified into acute and old fractures based on the presence or absence of bone marrow edema, respectively.
Benign vs malignant fracture	• Differentiation of benign and malignant fractures. • Benign fractures show horizontal band of marrow edema, concave appearance of posterior vertebral margin and lack of soft tissue mass. • Malignant fractures show almost complete involvement of vertebral body, convex posterior margin and associated soft tissue mass.

## Indications of spinal MRI

The main indications of MRI in spinal trauma include [2–4]:

1. Radiographic and/or CT scan findings suggestive of ligamentous injury, such as prevertebral hematoma, spondylolisthesis, asymmetric disc space widening, facet joint widening or dislocations, and inter-spinous space widening.
2. To look for epidural hematoma or disc herniation before attempting a closed reduction of cervical facet dislocations.
3. To identify spinal cord abnormalities in patients with impaired neurological status.
4. To exclude clinically suspected ligamentous or occult bony injuries in patients with negative radiographs.
5. To determine the stability of the cervical spine and assess the need for cervical collar in obtunded trauma patients.
6. To differentiate between hemorrhagic and non-hemorrhagic spinal cord injuries for the prognostic significance as the presence of hemorrhage significantly worsens the final clinical outcome.

According to American College of Radiology (ACR) appropriateness criteria, MRI of spine combined with CT scan is appropriate in the setting of acute spinal trauma if [5]:

1. National Emergency X-Radiography Utilization Study (NEXUS) or Canadian Cervical-Spine Rule (CCR) criteria are met and there are clinical findings of myelopathy.
2. NEXUS or CCR criteria are met and there are clinical or imaging findings to suggest ligamentous injury.
3. NEXUS or CCR criteria indicate imaging and the mechanically unstable spine is anticipated.

## Technical Considerations for MRI

The typical MRI protocol for spinal injury includes sagittal T1 weighted (T1W) and T2 weighted (T2W) spin echo sequences, and T2* weighted (T2*W) gradient recalled echo (GRE) sequence, and sagittal short tau inversion recovery (STIR) sequences, as well as axial T2W and T2*W GRE sequences. T1W images are mainly used for depiction of anatomy and osseous fractures. STIR images are very sensitive for detection of edema and is helpful in diagnosing the soft tissue and ligamentous injuries, particularly of the interspinous or supraspinous ligaments. Although fat-suppressed T2W images can also be used for detection of edema, STIR images provide more uniform fat suppression. T2W images are very good in detecting the cord edema, and T2*W GRE images are used to detect the hemorrhage in and around the cord [6]. Recently, diffusion tensor imaging (DTI) has been used to detect trauma related changes in the spinal cord which are not seen on conventional MRI technique [7, 8]. Ideally MRI should be performed within 72 hours of injury as the T2 hyperintensity produced by edema improves the conspicuity of the ligaments which are seen as low signal intensity in normal state [9]. Later on, resolution of the edema and hemorrhage reduces sensitivity of MRI to detect ligamentous injuries.

## Normal anatomy of the spine

The spine mainly consists of vertebrae stabilized by multiple ligaments including the anterior longitudinal ligament (ALL), posterior longitudinal ligament (PLL), ligamentum flavum, interspinous ligament, supraspinous ligament, and the apophyseal joint capsules [10]. Anatomy of the craniocervical junction is different from the rest of the spine and consists of many ligaments. However, only tectorial membrane, the transverse ligament, and the alar ligaments act as major stabilizers. While normal tectorial membrane and transverse ligament can be easily visualized on MRI, due to lack of contrast from adjacent tissues, the normal alar ligaments are difficult to be visualized [11].

## Three-column concept of spinal stability

Based on biomechanical studies, the vertebral column can be divided into three vertical parallel columns (i.e. anterior, middle and posterior columns) according to the Denis classification for the purposes of evaluating stability [12]. Spinal injury is usually classified as unstable when two contiguous columns are affected. The anterior column consists of ALL, anterior two-thirds of the vertebral body and anterior two-thirds of the intervertebral disc. The middle column consists of posterior one-third of the vertebral body, posterior one-third of the intervertebral disc, and PLL. The posterior column consists of everything posterior to the PLL including pedicles, facet joints and articular processes, ligamentum flavum, neural arch and interconnecting ligaments [13].

## Stable vs. unstable spinal injuries

**Fig. 1:**
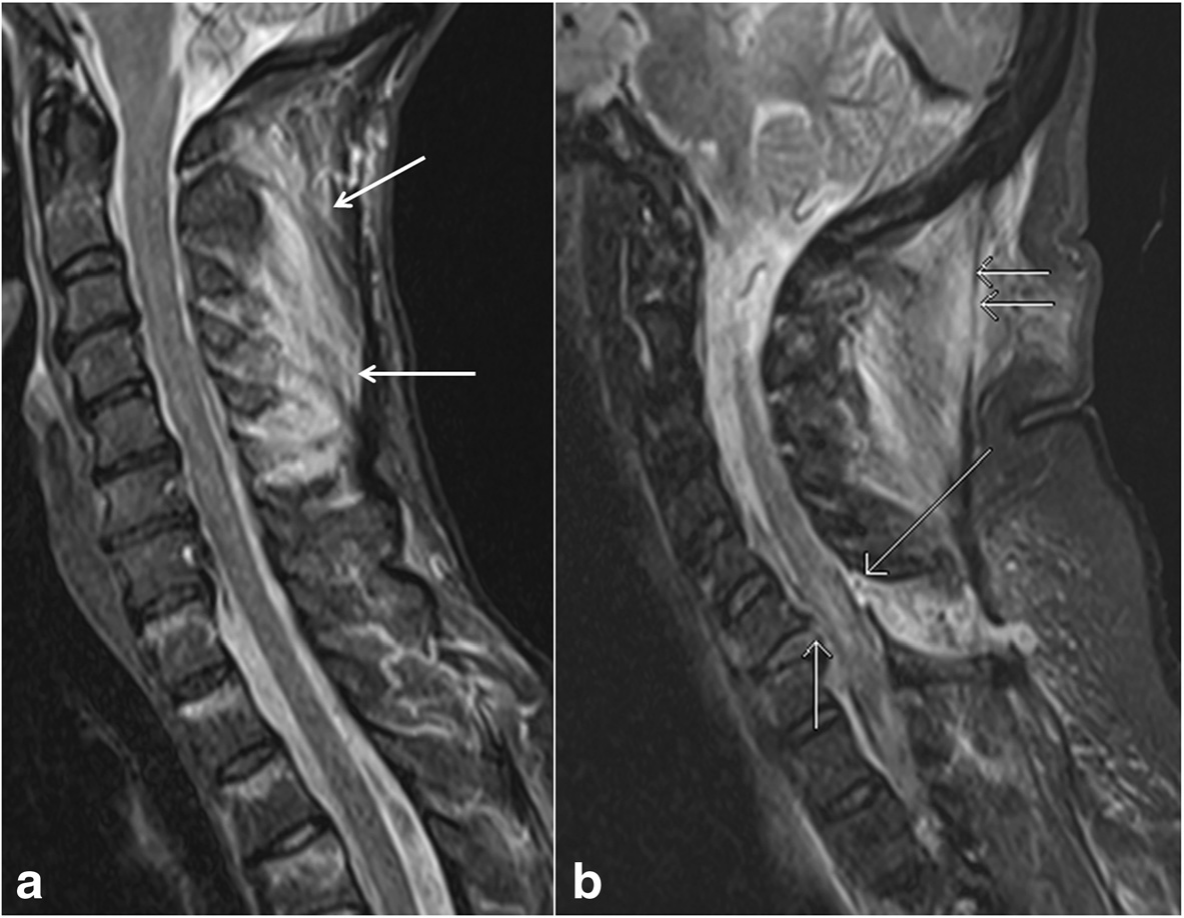
Sagittal short tau inversion recovery (STIR) image (**a**) of a patient shows a cervical spine injury involving interspinous ligaments (*arrows*), suggesting a stable single column injury. Sagittal STIR image (**b**) of a different patient with a cervical spine injury demonstrates a complete posterior longitudinal ligament tear (*short single arrow*), ligamentum flavum tear (*long single arrow*), and ligamentum nuchae tear (*short double arrows*), suggesting two column involvement and unstable nature of the injury

The most important radiological finding to suggest spinal instability is the involvement of two columns, based on the Denis classification as described above, which includes middle column in most cases (Fig. 1a, b). Other imaging findings of instability include translation of greater than 2 mm which indicates ligamentous injury, widening of the facet joints and interspinous space, disruption of the posterior vertebral body line, greater than 50 % loss of vertebral body height, and greater than 20 degrees of kyphosis. CT is sufficient to demonstrate most of these findings, but is relatively insensitive for the detection of ligamentous injuries. Thus, additional benefit of MRI is its ability to visualize ligamentous injuries responsible for instability [14–16].

## Types and mechanisms of ligamentous injury

**Fig. 2:**
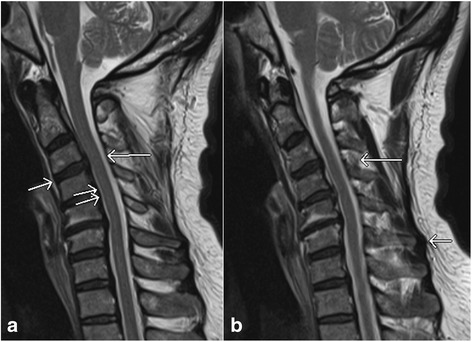
Sagittal T2 weighted image (**a**) shows normal anterior longitudinal ligament (*short single arrow*), posterior longitudinal ligament (*short double arrows*) and ligamentum flavum (*long arrow*). Sagittal T2 weighted image (**b**) shows normal wavy supraspinous ligament (*short arrow*), and normal striated interspinous ligament (*long arrow*)

Spinal ligaments are very important to maintain the normal alignment between vertebral segments under a physiologic load. Normal ligaments of the spine appear as low signal intensity bands on all the sequences (Fig. 2a). Notable exceptions are the interspinous ligament which may have striated appearance with low signal intensity areas interspersed with high signal intensity areas related to fat on T1W images, and the supraspinous ligaments which may demonstrate intermediate signal intensity on short TE pulse sequences owing to normal wavy appearance (Fig. 2b) [17].

**Fig. 3:**
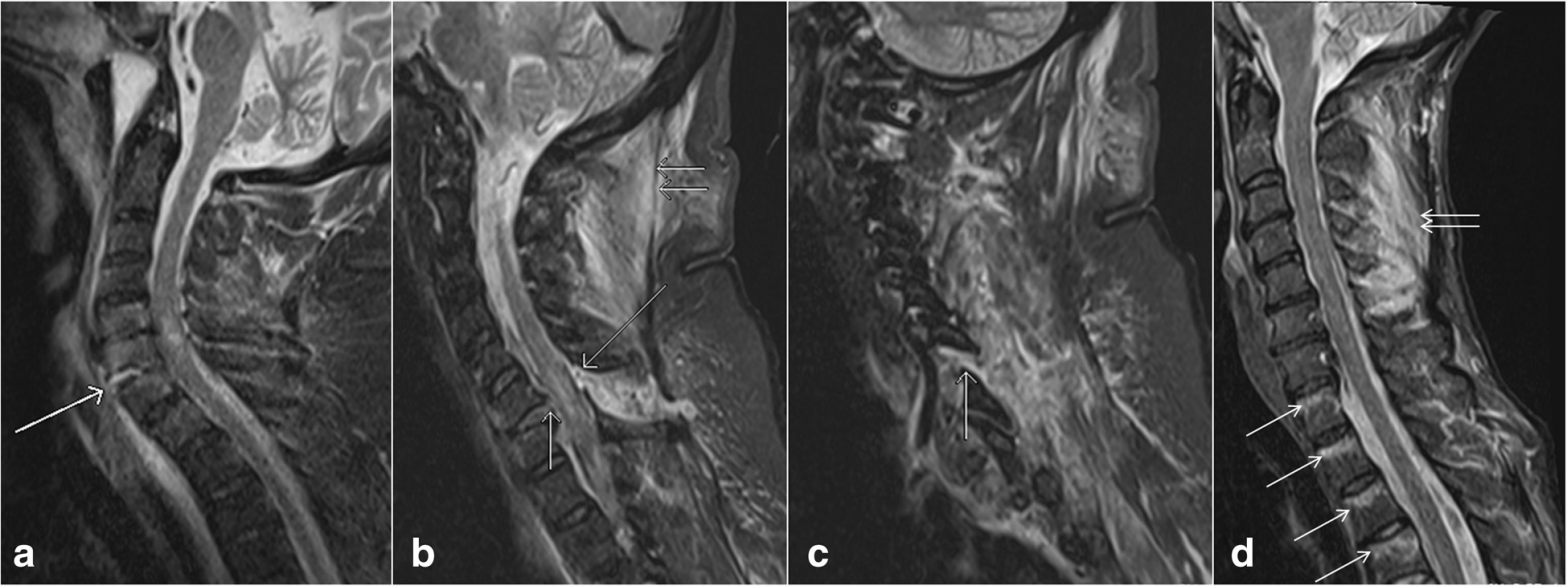
Sagittal short tau inversion recovery (STIR) images show complete anterior longitudinal ligament tear (*arrow*, **a**), complete posterior longitudinal ligament tear (*short single arrow*, **b**) and ligamentum flavum tear (*long arrow*, **b**), ligamentum nuchae tear (*short double arrows*, **b**), facet capsular injury (*arrow*, **c**), and interspinous ligament injury (*short double arrows*, **d**). Also note the presence of thoracic vertebrae contusions (*arrows*, **d**)

Ligamentous tears can be partial or complete. Partial tears are seen as high signal areas on STIR images related to edema and hemorrhage with varying degrees of intact fibers. Complete tears are seen as complete lack of intact fibers with high signal intensity on STIR images due to associated edema and hemorrhage [18]. Other types of ligamentous injuries include stripping of the intact ligament, and combined osseous and ligamentous injury. Types of ligamentous injury is usually related to the mechanism of the trauma. Hyperextension injuries usually result in damages to the anterior column or combined anterior and posterior columns and thus involving the ALL and PLL (Fig. 3a) [19]. However, hyperflexion injuries can also result in posterior column or combined posterior and middle columns injuries characterized by damages to ligamentum flavum, interspinous ligaments, supraspinous ligaments, facet joint capsules, and PLL (Fig. 3b, c, d) [17].

ALL is the main stabilizing ligament of the anterior column and seen as hypointense line anterior to vertebral bodies. An ALL injury appears as focal disruption of the hypointense signal on all the sequences with associated prevertebral edema best identified on STIR images. Normally PLL is seen as a hypointense line posterior to vertebral bodies. Similar to ALL injuries, injuries to PLL also appears as focal discontinuity of the hypointense line [20]. Both ALL and PLL are better identified when elevated from the normal attachments by intervertebral discs, fluid or the bones. Ligamentum flavum is seen to connect the lamina, best identified on the parasagittal images. Injury of the ligamentum flavum is usually associated with posterior element fractures and seen as focal discontinuity. Interspinous and supraspinous ligament injuries are characterized by increased signal in the interspinous spaces and tip of the spinous processes, respectively, on STIR images. Partial tears or sprain of these ligaments are more common than complete tears. Injuries to the facet joint capsule are seen as widening of the facet joint with increased fluid signal between the joint surfaces. Since ligaments are essential components of spinal columns, the presence of their injury can change a single column injury to a two column injury, thus upgrading a stable injury to an unstable injury [21].

The Spine Trauma Study Group developed another scoring and classification system named ‘The Thoracolumbar Injury Classification and Severity Score (TLICS).’ This newer system was devised based on three injury characteristics: radiographic injury morphology, integrity of the posterior ligamentous complex (PLC), and neurologic status of the patient, to provide an overall severity score, enabling stratification of patients into surgical and nonsurgical treatment groups [22].

## Acute traumatic disc herniation

**Fig. 4:**
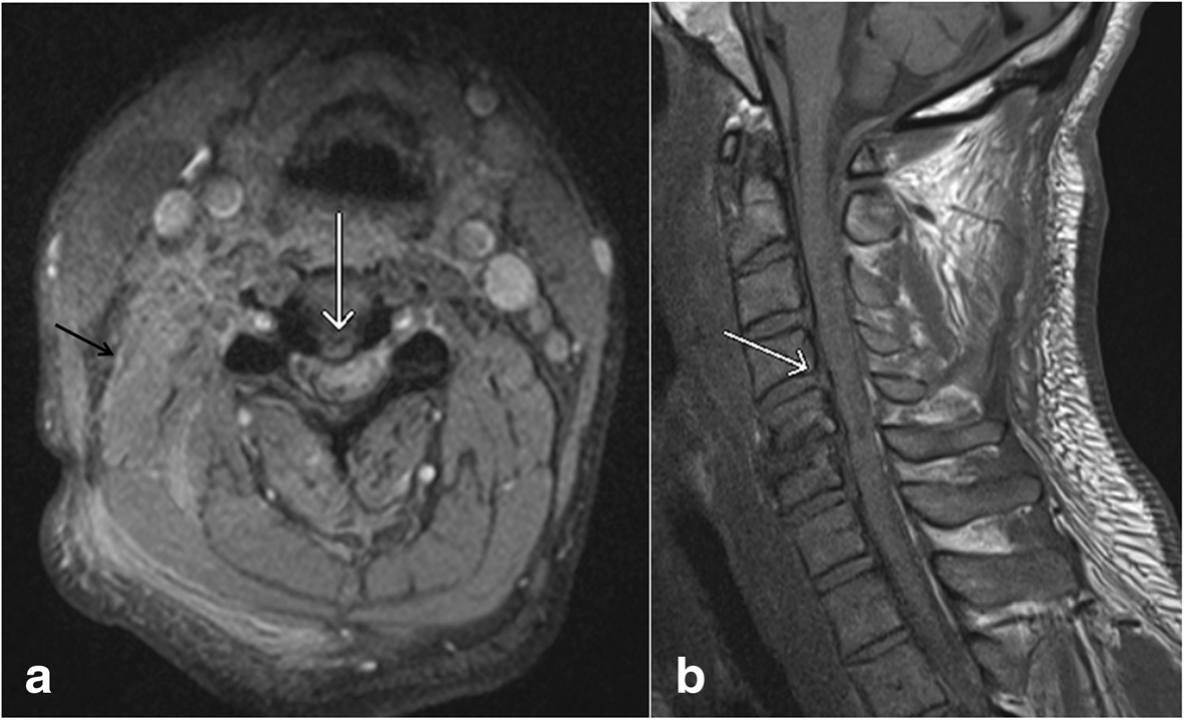
Axial gradient recalled echo (GRE) image (**a**) and sagittal T1 weighted image (**b**) show the presence of a small central disc herniation (*white arrows*). Also note the presence of paraspinal muscle edema (*black arrow*, **a**)

Traumatic disc herniations are most commonly associated with vertebral fracture dislocations and hyperextension injuries of the spine, and are caused by injuries to annulus fibrosus with nucleus pulposus herniation. On MRI, these can appear similar to non-traumatic disc herniations (Fig. 4a, b), and may cause compression of spinal cord leading to central cord syndrome in some cases [23]. MRI is better than CT in evaluating the traumatic disc herniations due to excellent contrast between disc, vertebral body and cerebrospinal fluid on appropriate pulse sequences. Additionally, multiplanar MRI is very helpful in evaluating large disc extrusions and sequestrated disc fragments before closed reduction of spinal dislocations [24]. Undetected disc herniations can cause new or worsening cord injury with progressive neurological deficits. Disc injuries without herniations are characterized by asymmetric widening or narrowing of the disc with abnormal signal related to edema. Histologically, these changes may be related to rupture of annulus fibrosus with hematoma [25].

## Extra medullary hemorrhage and fluid collections

**Fig. 5:**
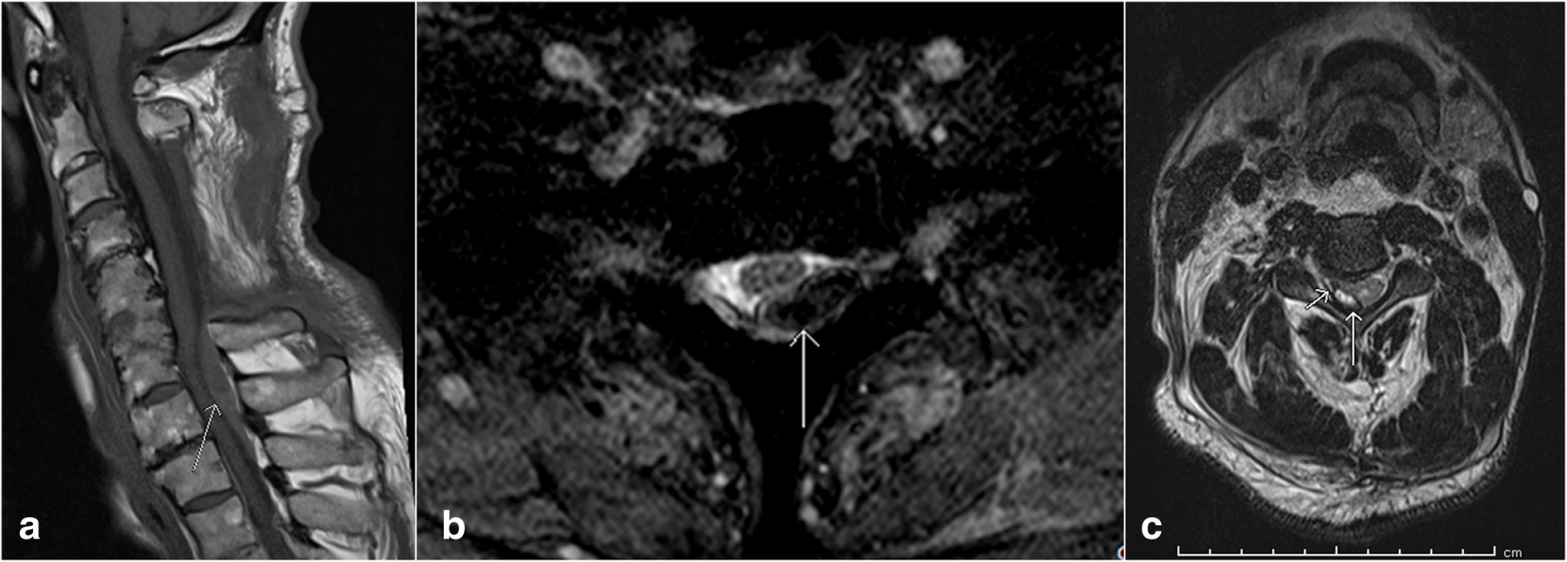
Sagittal T1 weighted image (**a**) and axial gradient recalled echo (GRE) image (**b**) show the presence of epidural hematoma (*arrows*), and axial T2 weighted image (**c**) shows subdural hematoma (*long arrow*) deep to the dura (*short arrow*)

Extradural hematoma is the most common type of extra medullary collections in trauma patients. Subdural hematoma and subarachnoid hemorrhage are uncommon. Pseudomeningoceles and extradural fluid collections due to dural tear are other uncommon sequelae of spinal trauma. Although CT can show the various types of hematomas in the spinal canal, due to beam hardening artifacts in CT and better soft tissue contrast resolution in MRI, MRI is the modality of choice for imaging of these entities. Epidural hematomas usually appear isointense to slightly hyperintense on T1W images and hyperintense on T2W images (Fig. 5a, b). Entire craniocaudal extent of the hematoma can be easily evaluated on sagittal MRI. Similar to epidural hematomas, subdural hematoma and subarachnoid hemorrhage show collections with varying signal intensities in the subdural (Fig. 5c) and subarachnoid spaces, respectively [26, 27].

## Vascular injuries

**Fig. 6:**
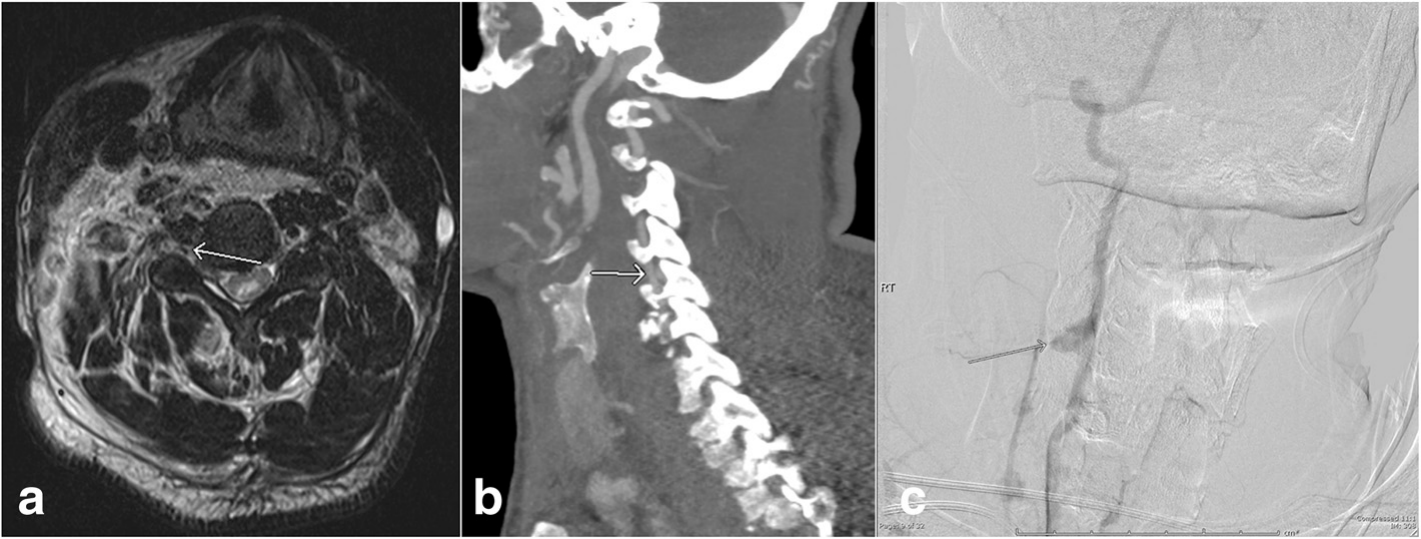
Axial T2 weighted image (**a**) shows the presence of post traumatic vertebral artery dissection with double lumen (*arrow*). Subsequent CT angiogram of the neck (**b**) confirms of the finding of vertebral artery injury (*arrow*). Follow-up angiography of the neck performed on the next day (**c**) shows the presence of pseudoaneurysm (*arrow*)

Vascular injuries can be caused by both blunt and penetrating trauma. In blunt vascular injuries in the neck, vertebral arteries are more commonly involved than carotid arteries. Although asymptomatic unilateral injuries are of less clinical significance, they can lead to cerebral and cerebellar infarctions, especially when bilateral [28]. The Denver screening criteria has been used to identify the patients at risk for vascular injuries and includes C1–C3 fractures, fracture of the cervical spine extending into a foramen transversarium, cervical spine subluxation, Le Fort II or III facial fractures, basilar skull base fractures involving the carotid canal, diffuse axonal injury, and expanding neck hematoma [29]. In case of thoracolumbar spine trauma, injuries to the aorta and its branches can occur. The imaging findings of vascular injuries include minimal intimal injury, visualization of intimal flap (Fig. 6a, b), pseudoaneurysm (Fig. 6c), dissection with intramural hematoma, complete occlusion, active extravasation, and arteriovenous fistula formation [29]. Most of the vascular injuries can be seen as irregularity or loss of normal flow void on long TE sequences such as T2W images. Fat-suppressed T1W images are better to identify the high signal intensity intramural hematoma associated with dissection. In equivocal cases, CT angiography or catheter angiography can be used for further evaluation of vascular injuries [30].

## Spinal cord injuries

**Fig. 7:**
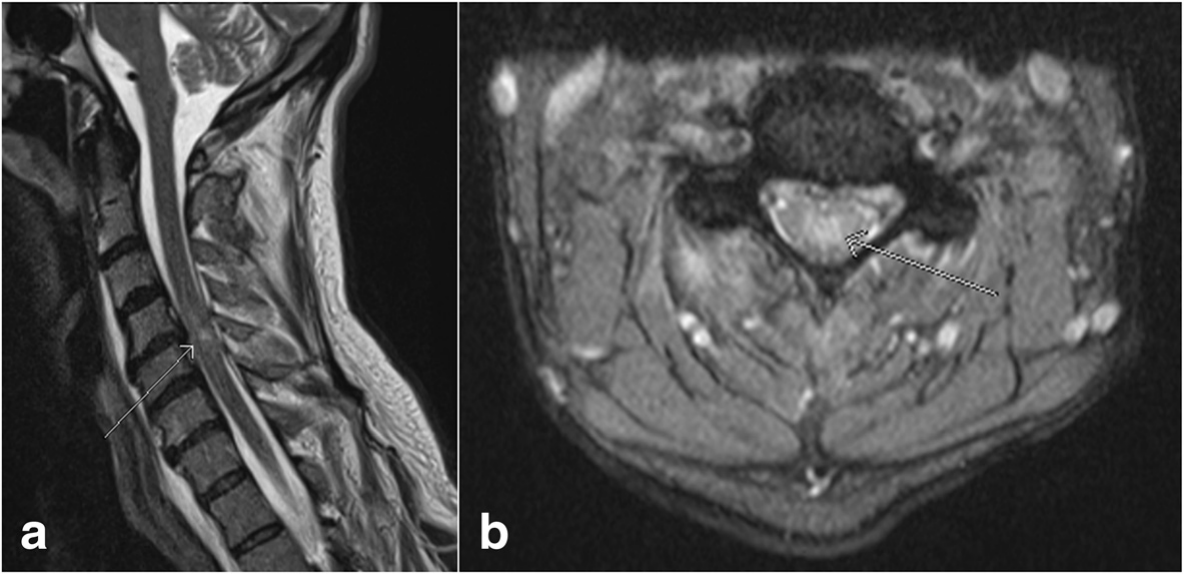
Sagittal T2 weighted image (**a**) and axial gradient recalled echo (GRE) image (**b**) show the presence of nonhemorrhagic contusion in the spinal cord (*arrows*)

**Fig. 8:**
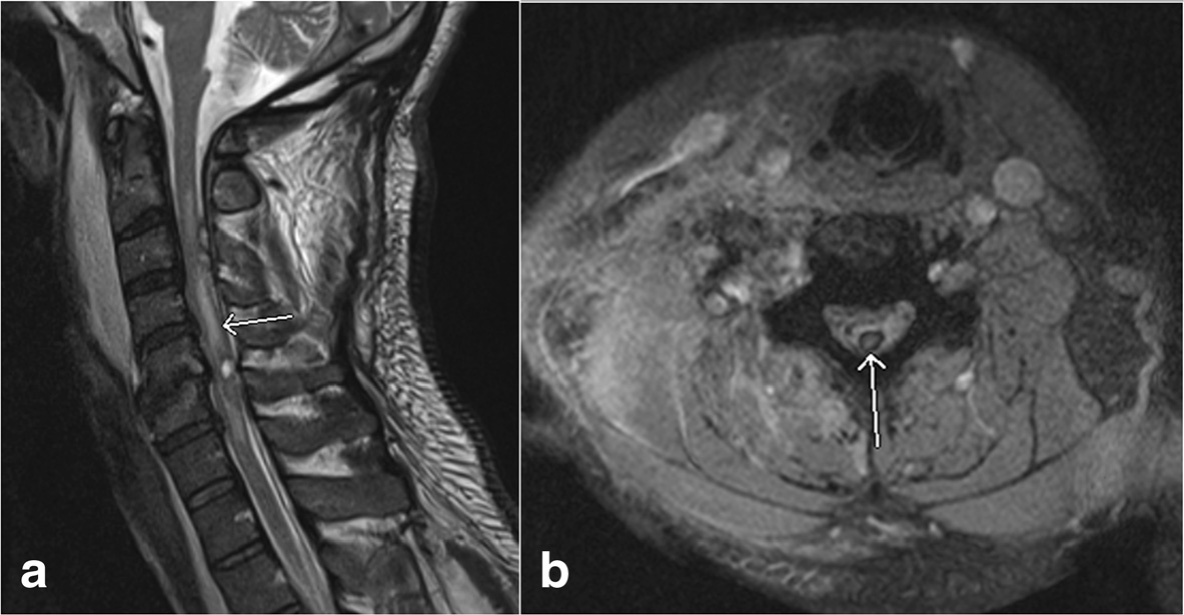
Sagittal T2 weighted image (**a**) and axial gradient recalled echo (GRE) image (**b**) show the presence of hemorrhagic contusion (*arrow*, **a**) in the spinal cord characterized by susceptibility artifact on GRE image (*arrow*, **b**)

Clinically, the extent of spinal cord injury is defined by the American Spinal Injury Association (ASIA) Impairment Scale (which is modified from the Frankel classification) using the following categories [31–33]: A = Complete – no sensory or motor function is preserved in sacral segments S4-5; B = Incomplete – sensory, but not motor, function is preserved below neurologic level and extends through sacral segments S4-5; C = Incomplete – motor function is preserved below the neurologic level, and most key muscles below the neurologic level have a muscle grade < 3; D = motor function is preserved below the neurologic level, and most key muscles below the neurologic level have a muscle grade ≥ 3; E = Normal. In MRI assessment of spinal cord injury, the axial and sagittal T2W images, and T2*W GRE images are particularly useful. Most common MRI findings of cord trauma include abnormal hyperintense T2 signal suggesting cord edema (Fig. 7a, b), hypointense signal depicting hemorrhage which is best seen on GRE images, and a mixture of edema and hemorrhage (Fig. 8a, b) [34]. Although neurological function at the presentation remains the single best predictive factor for long term prognosis, presence of cord hemorrhage has been described as the most important findings associated with poor prognosis. Other findings of prognostic value include the extent of cord hematoma and cord edema, and spinal cord compression by extra-axial hematoma [35]. Acute traumatic central cord syndrome, which is characterized by disproportionately greater upper extremities motor function impairment than in the lower extremities with bladder dysfunction and sensory loss below the level of injury, has been reported more frequently in hyperextension injuries in older patients with degenerative changes in the spine. Due to narrowed spinal canal, osteophytes or buckled ligamentum flavum may result in injuries to the central grey matter including the central portions of corticospinal tracts of the cervical cord [36–38].

## Other osseous and soft tissue injuries

**Fig. 9:**
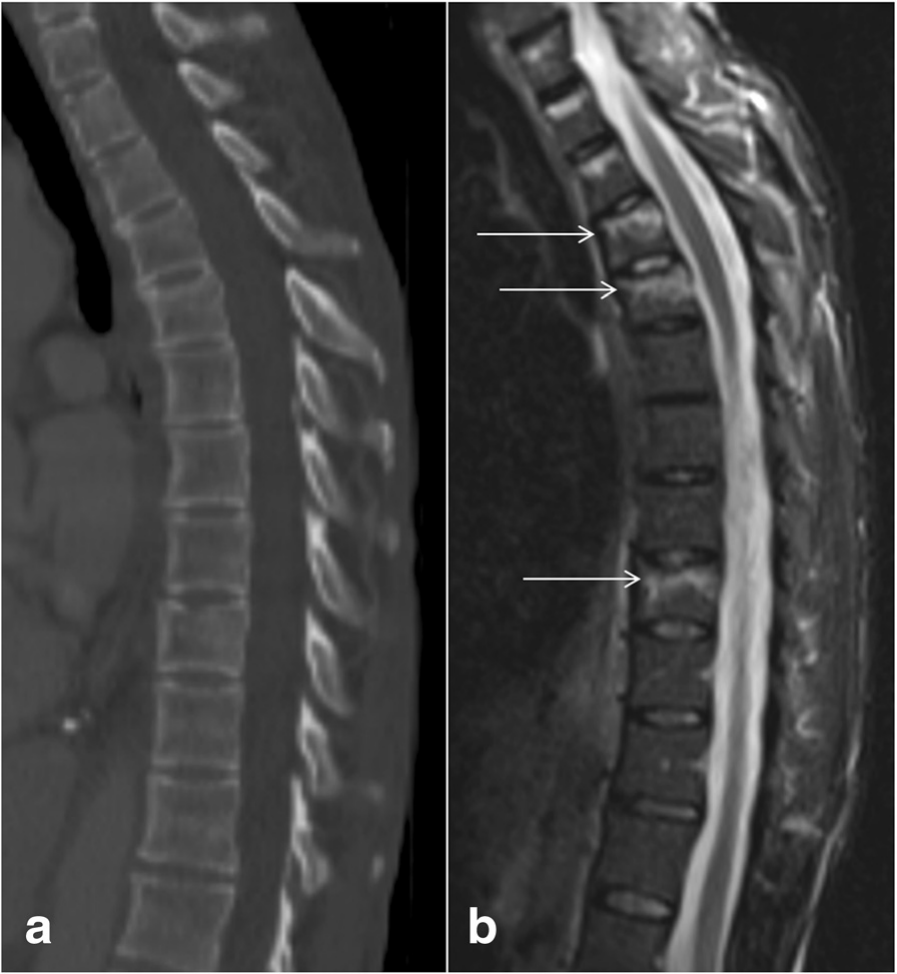
Sagittal CT image in the bone window (**a**) did not show any CT evidence for a fracture in this trauma patient. However, sagittal short tau inversion recovery (STIR) image (**b**) shows bone marrow edema in the superior aspect of multiple vertebrae (*arrows*) suggesting bone contusions

**Fig. 10:**
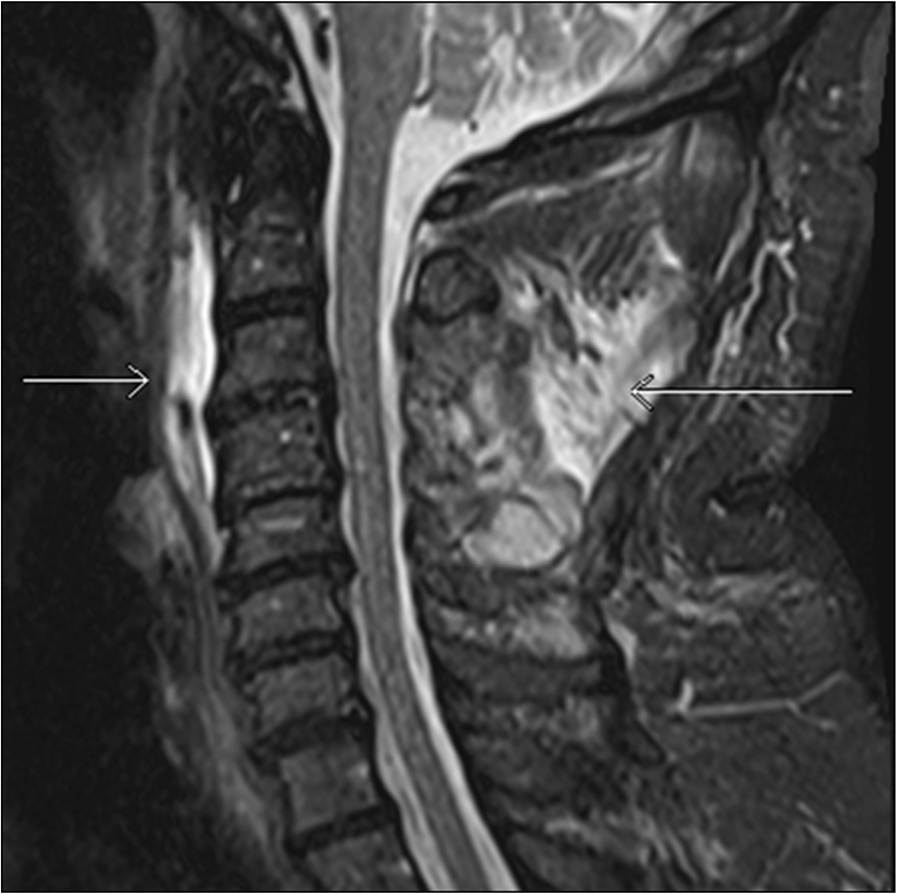
Sagittal short tau inversion recovery image shows prevertebral edema/hemorrhage (*short arrow*) and paraspinal muscle edema suggesting muscle injuries (*long arrow*)

Osseous injuries with little apparent morphologic changes such as compression and cortical break are difficult to be diagnosed with CT. MRI is very sensitive for detection of these occult osseous injuries by showing marrow edema and hemorrhage as hyperintense signal on fluid-sensitive sequences such as STIR (Fig. 9a, b) [39]. Prevertebral soft tissue injuries can occur and may demonstrate abnormal thickening. This finding is usually related to edema and hemorrhage and is a sensitive indicator of other serious injury to the spine (Fig. 10) [40]. This is usually seen in association with hyperextension injuries and vertebral body fractures, and may suggest underlying ALL injuries. Paraspinal muscles, nerves and other soft tissue injuries can also occur with trauma, either in isolation or associated with other injuries. Muscle strain will have edema seen as high signal intensity on STIR images (Fig. 10), while muscle hemorrhage will have heterogeneous signal intensity depending upon the presence of varying degrees and stages of hemorrhage mixed with edema. Usually, isolated muscle injuries are not clinically significant, but they can help to explain the cause of pain in the absence of other significant injuries.

## Old vs acute vertebral fracture

**Fig. 11:**
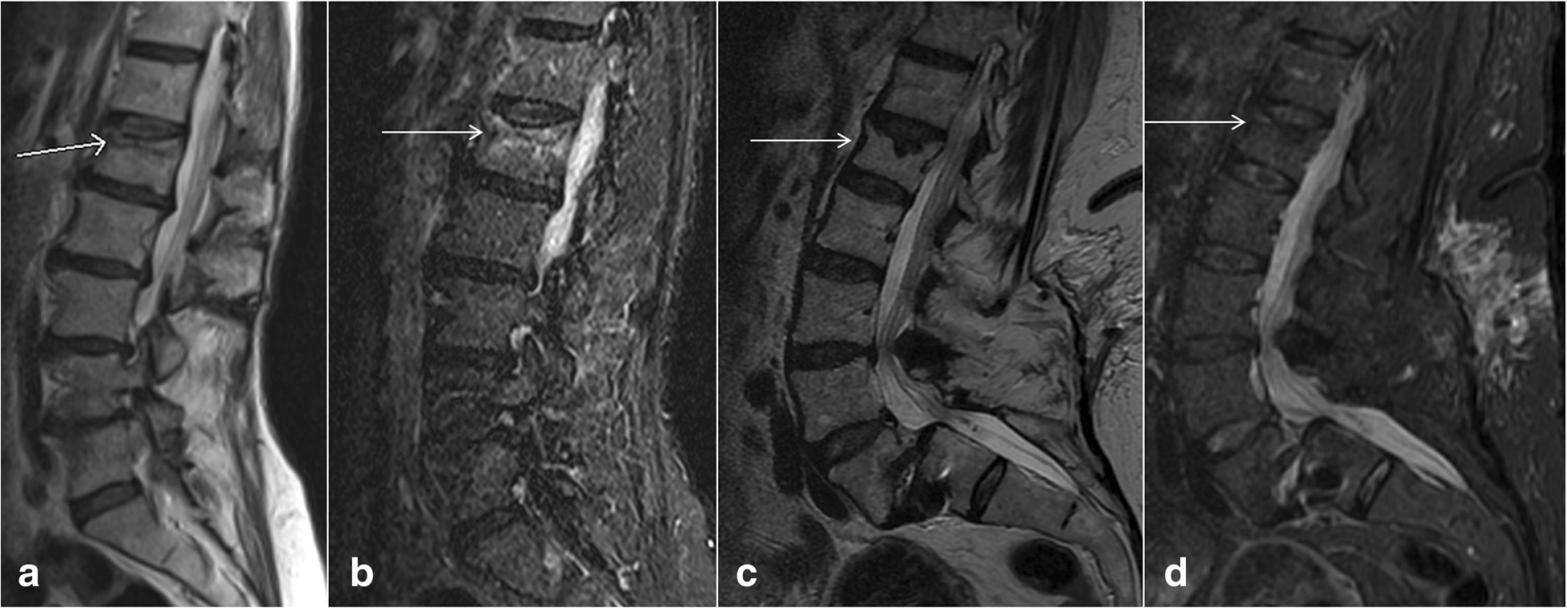
Sagittal T1 weighted image (**a**) and sagittal short tau inversion recovery (STIR) image (**b**) of a patient with an age-indeterminate fracture on CT showed bone marrow edema, suggestive of an acute vertebral body injury. Sagittal T1 weighted image (**c**) and sagittal STIR image (**d**) of a different patient with an age-indeterminate vertebral body fracture on CT showed no evidence of bone marrow edema, suggestive of a chronic injury

Compression vertebral fractures are very common, especially in the elderly, and are usually osteoporotic in etiology. Spine radiographs and CT are usually the initial diagnostic modalities for detection of osteoporotic vertebral compression fractures. Although review of prior images, history of recent trauma combined with physical examination findings and presence of soft tissue hematoma can be helpful to differentiate acute from chronic fractures, it may not always be possible to differentiate between the two. As acute fractures will cause bleeding and edema increasing the local water content, MRI can be very helpful by showing the bone marrow as low signal on T1W images and high signal on T2W and STIR images (Fig. 11a, b). Soft tissue edema associated with acute compression fractures can also be an important differentiating clue. The chronic fractures will show fatty marrow as high signal on T1W and T2W images without marrow edema (Fig. 11c, d) [41, 42].

## Benign osteoporotic fracture vs malignant fracture

**Fig. 12:**
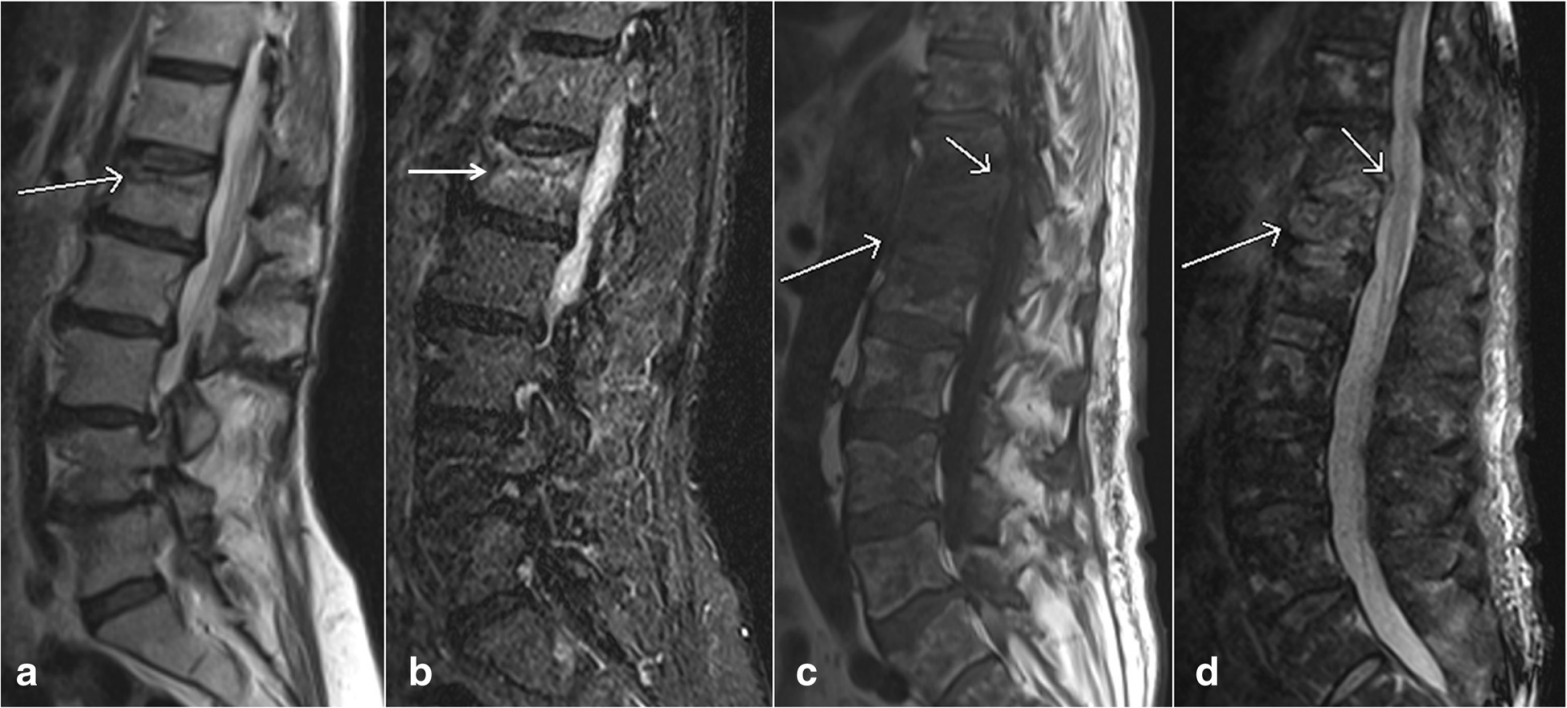
Sagittal T1 weighted image (**a**) and sagittal T2 weighted image (**b**) show a compression fracture of L1 vertebral body with a horizontal band of bone marrow edema in the superior half of vertebra without associated soft tissue mass, suggesting a benign compression fracture. Sagittal T1 weighted image (**c**) and sagittal T2 weighted image (**d**) of a different patient with multiple metastases show compression fractures with diffuse involvement of vertebral body and associated epidural soft tissue mass. Note abnormal bone marrow signal intensities also seen in other vertebral bodies, reflecting diffuse metastatic disease

Differentiating acute osteoporotic fractures from acute pathological fractures caused by metastases and other primary malignancies is a commonly encountered dilemma in the clinical practice. This is especially more important in deciding the appropriate treatment in patients with known primary tumor and no other known metastasis. MRI findings favoring acute osteoporotic compression fractures would include horizontal band of abnormal signal intensity separated by a straight line from the normal fatty marrow, relative lack of involvement of posterior elements, and angulated and concave appearance of the posterior vertebral margin (Fig. 12a, b). In contrast, pathological fractures due to malignancy on MRI are characterized by involvement of the entire vertebral body by abnormal bone marrow edema, extension into posterior elements, convex appearance of the posterior vertebral wall, involvement of the surrounding soft tissue, and the presence of other bony lesions (Fig. 12c, d) [43]. Diffusion weighted imaging also has been shown to be useful in differentiating these two by showing restricted diffusion in malignant pathological fractures. This has been attributed to the high cellularity and high nucleocytoplasmic ratio in rapidly dividing tumor cells. In patients at risk of having metastatic disease, limited follow-up MRI in 6–8 weeks is recommended to demonstrate partial or complete resolution of bone marrow edema in a situation where acute osteoporotic fractures are considered likely. In comparison, malignant pathological compression fractures will remain unchanged or progress on follow-up examinations. Alternatively, biopsy can be performed in select cases for prompt and accurate treatment planning.

## Pitfalls of MRI

**Fig. 13:**
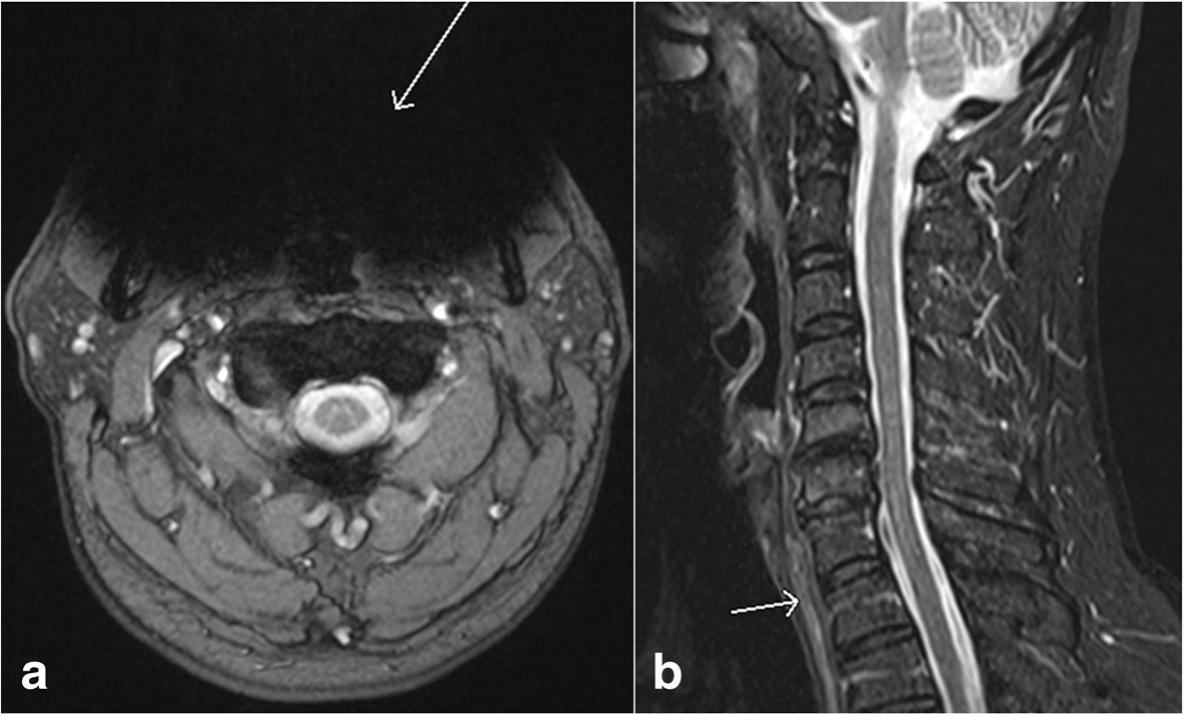
Axial gradient recalled echo (GRE) image (**a**) shows marked susceptibility artifact (*arrow*) due to dental implants which partially obscures visualization of the prevertebral soft tissues. Sagittal T2 weighted image (**b**) shows the presence of fluid in the esophagus (*arrow*) which may mimic prevertebral soft tissue edema

**Fig. 14:**
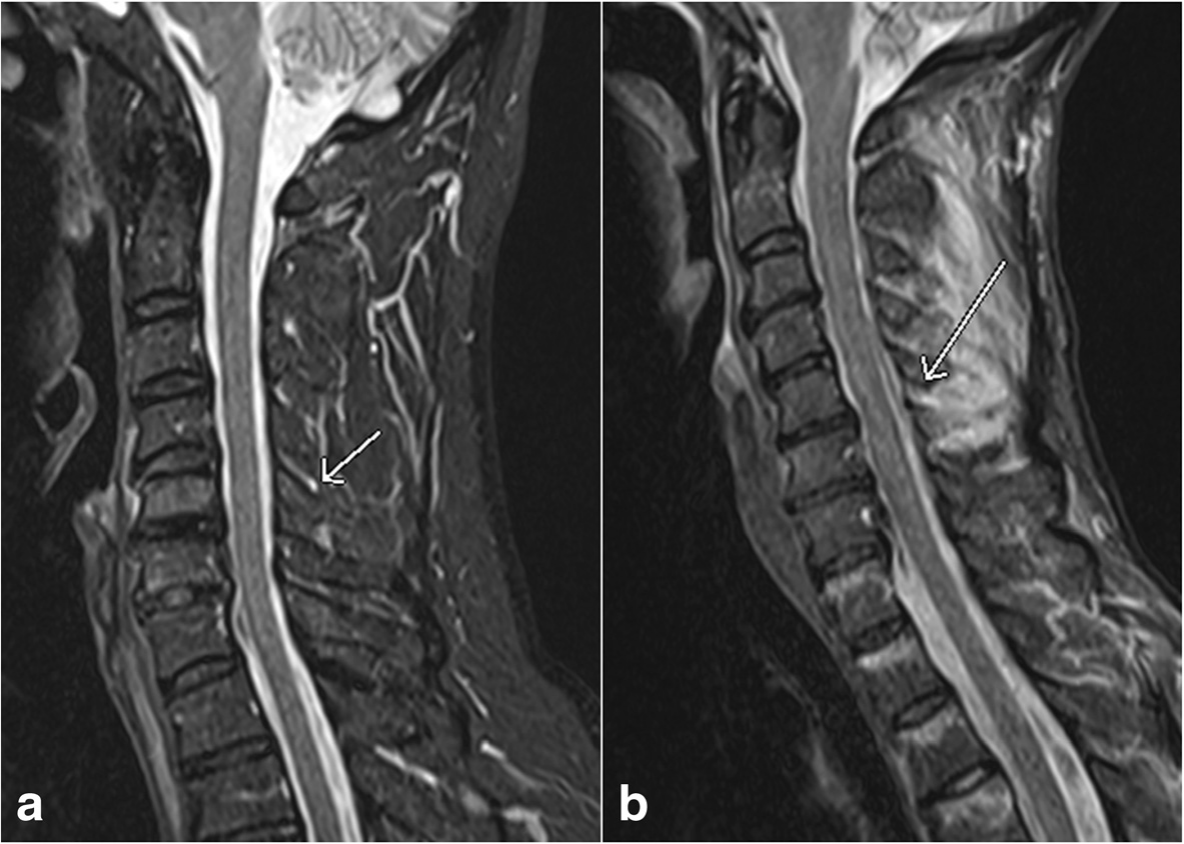
Sagittal short tau inversion recovery (STIR) image of cervical spine (**a**) shows the presence of blood vessels in the interspinous space which are seen as well defined high signal (*arrow*). This is in contrast to ill-defined high signal intensity (*arrow*) in the interspinous ligament on a sagittal STIR image (**b**) of a different patient who sustained acute interspinous ligament injury

There are few limitations of MRI in the evaluation of spinal trauma. Susceptibility artifacts due to metallic hardware for spinal fusion and dental implants can degrade the image quality especially on GRE sequence (Fig. 13a). Susceptibility artifacts can be reduced by using the spin echo sequences, short TE which allows less time for dephasing and reduces signal loss, large receiver bandwidth, STIR rather than chemically selective fat suppression, and swapping the phase-encode and frequency-encode directions [44]. Saturation pulses used in MRI can sometimes mask the prevertebral hematoma [15]. Fluid in esophagus (Fig. 13b) and incomplete suppression of the prevertebral fat can sometimes simulate prevertebral edema. Prominent veins in the interspinous region demonstrating high signal on STIR images should not be confused with edema associated with interspinous injuries which appear as ill-defined area of high signal, while the veins appear as well-defined linear areas of high signal (Fig. 14a, b). The sensitivity of MRI is also lower than CT for detecting fractures of the posterior elements due to minimal edema associated with avulsion injuries, and to injuries of the craniocervical junction [45].

## Conclusions

In conclusion, MRI is more sensitive than other imaging modalities in the diagnosis of soft tissue and spinal cord injuries. While CT is considered adequate for determination of stable vs unstable spinal injuries, MRI can offer additional help due to its ability to better diagnose ligamentous injuries when compared with CT. MRI is also helpful in predicting the prognosis by demonstrating the hemorrhagic and non hemorrhagic cord injuries.

## Abbreviations

ACR, American College of Radiology; ALL, Anterior Longitudinal Ligament; CCR, Canadian Cervical-Spine Rule; CT, Computed tomography; DTI, Diffusion Tensor Imaging; GRE, Gradient Recalled Echo; MRI, Magnetic Resonance Imaging; NEXUS, National Emergency X-Radiography Utilization Study; PLC, Posterior Ligamentous Complex; PLL, Posterior Longitudinal Ligament; STIR, Short Tau Inversion Recovery; TE, Echo Time; TLICS, Thoracolumbar Injury Classification and Severity Score
